# Unusual Case of Malignant Struma Ovarii and Cervical Thyroid Cancer Preceded by Ovarian Teratoma: Case Report and Review of the Literature

**DOI:** 10.1155/2019/7964126

**Published:** 2019-03-17

**Authors:** Elias G. Tzelepis, Elena Barengolts, Steven Garzon, Joseph Shulan, Yuval Eisenberg

**Affiliations:** ^1^Division of Endocrinology, Diabetes & Metabolism, University of Illinois at Chicago, Chicago, IL, USA; ^2^Department of Pathology, University of Illinois at Chicago, Chicago, IL, USA

## Abstract

**Objective:**

To present a rare case of malignant struma ovarii (MSO) and synchronous thyroid cancer, review the medical literature, and present the latest trends in management.

**Methods:**

The case of a woman with MSO and concomitant thyroid cancer is presented, including clinical presentation, treatment, and follow-up care. A search of the English-language literature was conducted using MEDLINE and Google Scholar data bases.

**Results:**

We found 10 publications (one abstract) describing 10 patients with MSO and concomitant thyroid cancer. Six additional patients were reported by a study that analyzed the SEER (cancer registry) database. The median age of women was 42 years, with the majority of them presenting with abdominal symptoms. Histologically, most tumors were papillary carcinomas in both organs. In 5 patients, there was extrathyroidal tumor extension at time of surgery.

**Conclusion:**

MSO can occasionally coexist with highly aggressive eutopic thyroid cancer. Although this concurrence is even rarer than MSO, clinicians should routinely investigate for possible synchronous thyroid cancer in all cases of MSO and also consider aggressive postoperative treatment including thyroidectomy and radioiodine ablation therapy in cases of MSO.

## 1. Introduction

Struma ovarii refers to a germ cell tumor that is composed of at least 50% thyroid tissue [1, 2]. These tumors account for about 2-3% of all ovarian tumors [1–3]. They are relatively rare, with no more than 200 cases reported in the literature [4].

Most struma ovarii are benign, with malignant transformation occurring in only 5% of these tumors [5, 6]. Malignant struma ovarii (MSO) coexisting with primary thyroid carcinoma is extremely rare, with only a handful of cases reported in the literature [2, 7, 8]. The synchronous development of primary thyroid carcinoma and MSO is not fully understood. Investigations including morphological, immunohistochemical, and molecular analysis suggest that synchronous, albeit distinct, primary tumors in the ovary and cervical thyroid may occur [8, 9]. A hypothesized “field cancerization” and early genomic instability may explain multifocality in all thyroid-type tissue. Some experts, therefore, recommend routine imaging of the thyroid gland for coexisting carcinoma in all patients with high risk MSO [8]. Supporting evidence for this approach also comes from a population-level analysis reporting an approximately 9% prevalence of synchronous or metachronous aggressive thyroid cancers in a series of 68 patients with MSO [10].

Given the overall rarity of MSO and its possible concurrence with thyroid cancer, the optimal management of MSO is controversial, especially when the tumor is not metastatic [11–13]. Areas of contention include aggressiveness of therapy, prophylactic thyroidectomy, and radioactive iodine (RAI) [7, 8, 13, 14]. Here, we present the case of a woman with MSO and concomitant thyroid carcinoma, along with a literature review of similar cases reported in the English literature highlighting the current trends in management of this rare malignancy.

## 2. Clinical Case

A 32-year-old nulliparous female presented to the endocrinology clinic for further evaluation and treatment of a recently diagnosed MSO. Five years prior to this evaluation, she underwent bilateral ovarian cystectomy for complex ovarian masses which were histologically consistent with mature teratomas. Over the ensuing four years, serial ultrasonography showed progressive interval growth of the bilateral cystic masses, especially of the left adnexal mass growing from 4.3 cm to 12.9 cm in approximately 4 years.

**Figure 1:**
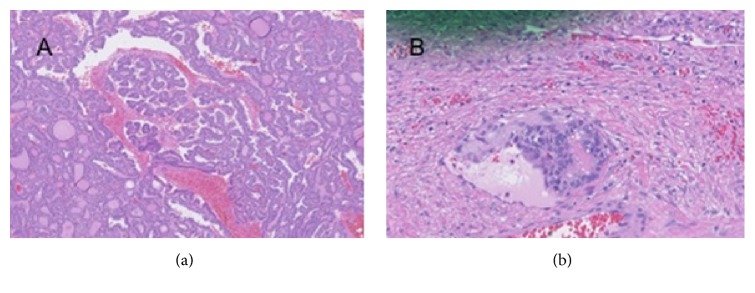
(a) Papillary thyroid carcinoma arising in struma ovarii. (b) Vascular invasion of malignant struma ovarii.

Five months prior to her endocrinology evaluation, she underwent exploratory laparotomy with left salpingo-oophorectomy, right ovarian cystectomy, lysis of adhesions, and partial omentectomy. Pathology showed a 6.0 cm malignant struma ovarii, with predominantly follicular variant papillary thyroid carcinoma in the left ovary (Figure 1(a)). The tumor, classified as pT1a pNx; FIGO IA, was organ confined but with lymphatic/vascular invasion (Figure 1(b)). The omentum was free of tumor, and in the right ovary a hemorrhagic corpus luteum cyst was identified. Postoperatively she underwent an I-123 whole body scan which did not identify any metastatic disease. Specifically, there was no abnormal uptake in the adnexae or the abdomen, although this was likely of limited utility given the intact thyroid gland.

When seen in the endocrinology clinic, she had no abdominal complaints. Physical exam was unremarkable, including a normal neck exam without palpable thyroid enlargement or nodularity and with a soft abdomen without palpable masses or ascites. Family history was significant for cervical and lung cancer in her mother as well as the maternal grandmother with breast and bone malignancies.

**Figure 2:**
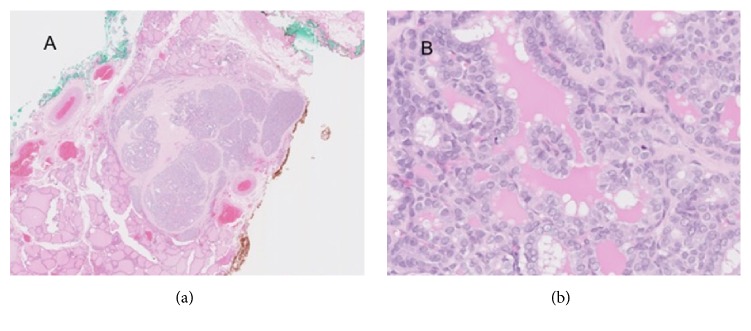
(a) Well circumscribed nodule composed of papillary thyroid carcinoma with solid, follicular, and papillary architecture surrounded by fibrosis. (b). Areas with papillary architecture with cytologically enlarged nuclei with grooves and open chromatin.

Laboratory testing showed TSH 2.98 mcIU/ml (0.35-4.0), FT4 16.7 pmol/L [1.3 ng/dL (0.6-1.7)], FT3 49.1 pmol/L [3.2 ng/dL (87-178)], thyroglobulin antibody negative, (<1.0) thyroglobulin 108.5 ng/mL (1.3-31.8). A thyroid ultrasound showed a mixed hypo- and isoechoic 0.8 x 0.4 x0.7 cm nodule with several echogenic punctate foci consistent with microcalcifications. Fine needle aspiration biopsy of the nodule was interpreted as atypia of undetermined significance (Bethesda III). Given the clinical history of MSO, indeterminate cytopathology, and patient preference, the decision was made to perform total thyroidectomy. Pathology revealed a 0.6cm papillary thyroid carcinoma with no lymphovascular invasion (Figure 2). There was, however, a single focus of extra thyroidal extension (pT3). Her postoperative course was complicated by hypoparathyroidism with symptomatic hypocalcemia which resolved with aggressive replacement of calcitriol and elemental calcium. Thyroglobulin levels were 9.5 ng/ml with negative thyroglobulin antibodies at two months after thyroidectomy. She was discharged on calcitriol 0.25mcg BID and calcium carbonate 2500mg TID (3000mg elemental calcium). She was started on liothyronine 25mcg PO daily, in anticipation of planned RAI therapy.

Three months later, I-123 WBS/SPECT/CT revealed multiple avid foci in the neck suggestive of lymph node metastasis and residual thyroid tissue, as well as one faint focus in the mid-abdomen possibly representing a metastatic mesenteric lymph node. Ultrasonography failed to reveal abnormal cervical lymph nodes. Considering these findings in total, it was decided to proceed with radioactive iodine therapy and she received 184.3 mCi of I-131 therapy. Post-therapy whole body scan revealed successful targeting of radioiodine therapy to thyroid tissue in the neck, and no convincing abnormal focal uptake in abdomen or pelvis to suggest radioiodine avid metastatic disease. Thyroglobulin levels post-RAI ablation decreased to 0.5ng/ml and subsequently to 0.1ng/ml upon later testing. BRAF mutational analysis testing for the V600E mutation was negative for both ovarian and thyroid papillary carcinomas. Patient also had testing for TERT promoter mutations, which were negative. Post-RAI ablation, patient was switched to levothyroxine 200mcg daily with subsequent titration to 225 mcg daily to achieve suppressed TSH. Hypocalcemia eventually resolved, and she was transitioned off calcitriol and calcium carbonate supplementation. A year after RAI ablation, the patient underwent rhTSH stimulated WBS which did not show any evidence of thyroid cancer recurrence. The most recent thyroid ultrasound (12/2017) showed stable benign appearing lymph nodes, and no evidence of residual or recurrent thyroid tissue (thyroglobulin level of 0.1 ng/ml). A pelvic surveillance MRI showed benign appearing cystic lesions at the right adnexa, but no evidence of metastatic disease. The patient provided written informed consent to publish her case.

## 3. Discussion

Malignant transformation of struma ovarii occurs in less than 5% of these ovarian tumors [5, 13]. Patients with MSO typically present with abdominal pain or an incidental ovarian mass, vaginal bleeding, menstrual cycle abnormalities, and symptoms of hyperthyroidism or rarely with ascites [13, 20–22]. Patients may occasionally present with symptoms of hyperthyroidism (5-8% of cases) [13] or elevated ovarian cancer markers (CA-125) [23]. Following diagnosis of differentiated thyroid cancer arising in struma ovarii, further work-up should include thyroid evaluation to exclude synchronous thyroid carcinoma.

Concurrence of MSO and intrathyroidal cancer is quite rare. In reviewing the literature, we found 10 publications, 9 case reports [2, 7–9, 14–16, 18], and one abstract [17], describing 10 patients with synchronous MSO and thyroid carcinoma (Table 1). Not shown in Table 1 are additional patients reported by two retrospective analyses of the Surveillance, Epidemiology, and End Results (SEER) database from 1973 to 2011 by Goffredo et al. [10] and Sisti et al. [24]. Goffredo et al. analyzed the entire database (SEER 18 Registries) whereas Sistri et al. only part of it (SEER 9 Registries). Although an overlap of the reported patients between the two analyses cannot be excluded, Goffredo et al. identified 6 patients (median age 36) with concomitant thyroid cancer out of 68 patients with MSO and Sisti et al. one patient with synchronous and one with metachronous thyroid cancer out of 21 patients with MSO. As most patients reported are from institutions outside the US (unlikely overlap with those reported by Goffredo et al.), the total number of reported MSO patients with coexisting thyroid cancer in the English literature is not greater than 17.

The median age of women described with concomitant MSO and intrathyroidal was 42 years (range 30-55), with the majority of them having ovarian masses larger than 5 cm. With exception of two patients [16, 19] who sought advice for cervical thyroid nodules (MSO diagnosed after thyroidectomy with radioiodine), the remaining patients had manifestations related to the abdomen. Size of thyroid tumors ranged from 0.2 to 1.7 cm. There were two patients with MSO size less than 1.0 cm. Histologically, most tumors were papillary thyroid carcinomas in both sites, with only one discordant case of a follicular thyroid cancer in MSO and papillary thyroid cancer in the cervical thyroid [7]. There were five patients with extrathyroidal tumor extension or metastasis to regional lymph nodes at time of thyroidectomy. Our patient had evidence of extrathyroidal tumor extension on thyroid pathology. She was also believed to have metastatic disease to cervical lymph nodes three months after thyroidectomy, based on thyroglobulin measures and nuclear imaging. Similarly, Goffredo et al. reported that the majority (two-thirds) of the patients with synchronous MSO and thyroid carcinoma had evidence of cancer extension outside the gland.

The relatively high prevalence (about 9%) of MSO coexisting with thyroid cancer reported by Goffredo et al. may support the notion for a true association and common pathogenic mechanism [10]. Evidence for a common pathogenesis for all papillary thyroid carcinomas was also provided by Schmidt et al. who found BRAF gene mutations in 4 out of 6 patients with MSO and in none of 9 patients with benign struma ovarii [25]. These data indicate that the development of MSO is associated with* BRAF* mutations of the type commonly seen in cervical thyroid papillary carcinoma. Although BRAF mutations in MSO were additionally found in other case reports [26] or small series of patients [27], we could not find any literature reports of BRAF mutations in both MSO and cervical thyroid cancer. Reporting differences in tumor expression of CK-19 in a patient with synchronous MSO and cervical thyroid cancer, Leong et al. [8] supported the notion of an autonomous existence of the two cancers and postulated an early genetic instability to explain the multifocal oncogenesis.

Concomitant MSO and thyroid cancer should be differentiated from papillary carcinoma of the cervical thyroid with ovarian metastasis, which is extremely rare [28]. Presence of teratomatous elements and normal thyroid epithelial tissue in the ovary is suggestive of MSO rather than metastatic thyroid cancer [8]. In this respect, ovarian MRI may help distinguish the two by revealing multiloculated ovarian cystic mass with a solid component and a variable signal intensity between locules (“stained glass imaging”) [29]. Our patient had known history of mature ovarian teratoma and the MRI was also consistent with multiloculated cysts.

The optimal management of MSO remains controversial [11–13]. As with most rare diseases, the natural history of MSO is not fully defined and there is a paucity of evidence-based clinical practice guidelines. The extent of ovarian surgery can be a subject of debate [30], especially when preservation of fertility is an issue. Preoperative ultrasonography may help differentiate benign from malignant pathologies and allow for ovarian sparing surgical planning if appropriate [31]. The most contentious aspects of MSO treatment include need for thyroidectomy and postoperative RAI in patients with no evidence of metastases [2]. Presence of metastases poses no therapeutic dilemma and all patients should be treated with thyroidectomy followed by RAI [2, 5, 32]. Given the possible coexistence of synchronous thyroid cancer, total thyroidectomy will exclude thyroid cancer, permit RAI for micrometastasis, and increase reliability of thyroglobulin as a marker for follow-up [7]. In six patients treated with thyroidectomy and RAI after resection of MSO, DeSimone et al. [13] reported no recurrence. In contrast, Jean et al. [33] reported a 21 % recurrence in 42 MSO patients treated only surgically.

Most authors [5, 13–15, 34–37] endorse an aggressive postoperative treatment with thyroidectomy followed by RAI therapy on the basis that RAI may reduce recurrence and possibly mortality [32, 33, 38] especially in cases of uncertainty about surgery completeness, in an analogy used to treat cervical thyroid cancer [1]. In two separate analyses of MSO patients reported in the literature, Devaney et al. [1] and DeSimone et al. [13] noted that more recurrences occurred in patients who were conservatively treated with surgery only than in those who received primary adjuvant therapy.

Risk stratification in MSO has been proposed as a means to help choose the appropriate treatment postoperatively in patients with no evidence of metastases at time of surgery [6]. Yassa et al. [6] suggested that MSO measuring over 2 cm, or with extraovarian extension, or aggressive histological features should be treated with thyroidectomy and RAI. However, tumor size criteria are not unanimously agreed upon by all authors, with some using cutoff tumor size of 1 cm [7]. Furthermore, measuring tumor size can be extremely difficult in cases in which the tumor is intermixed with teratomatous tissue [2]. Likewise, histological features of MSO were not found to correlate with clinical outcome in 26 patients with MSO followed up for a period ranging from 5 to 20 years [39]. Extraovarian tumor extension is generally considered an indication for aggressive treatment with thyroidectomy and RAI [2]. In our patient, the choice of postoperative treatment was indisputable as she had extraovarian tumor extension.

**Figure 3:**
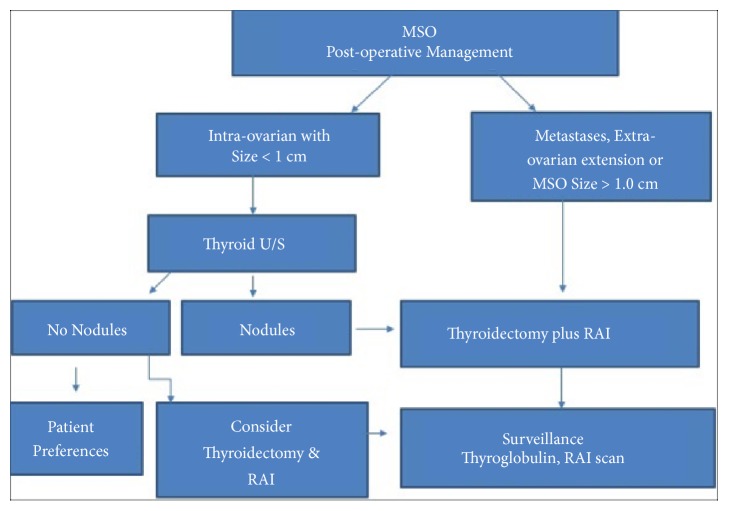
Algorithm summarizing current trends in postoperative management of malignant struma ovarii.

Given the rarity of the disease, the postoperative management of MSO will likely continue to be controversial and be based on results of small observational studies, rather than controlled studies. The present literature review provided a description of the coexistence of MSO and thyroid cancer, highlighting the relatively aggressive cervical thyroid cancer found in 45 % of reported cases (Table 1) which is in agreement with the findings of Goffredo et al. Considering the relatively high prevalence of these synchronous tumors in the SEER database and the generally increasing incidence of thyroid cancer [40], the clinician should be aware of the possible association and further investigate it in all patients with MSO. Despite the significant uncertainty and many unsettled issues about treatment, we propose an algorithm (Figure 3) for the postoperative management of MSO. Again, this algorithm summarizes the current trend of recommendations in the literature, which are based on small retrospective observational studies and are by no means firm and indisputable. As most authors [5, 13–15, 34–37], we endorse an aggressive postoperative management that should include thyroidectomy and RAI in all patients with MSO. Patient preferences might only be considered in cases in which the tumor is small and confined to ovary and there is no evidence of thyroid nodules. Ultrasonographic detection of thyroid nodules postoperatively will simply reinforce and justify this aggressive approach.

## 4. Conclusion

MSO, a rare malignant tumor arising from struma ovarii, can occasionally coexist with thyroid cancer. The present literature review supports the notion that the concurrent cervical thyroid tumors are usually aggressive cancers. Clinicians should be aware of this association and routinely investigate for thyroid nodules in all cases of MSO. In view of this association, we endorse an aggressive postoperative management of all MSO cases that includes thyroidectomy and RAI.

## Figures and Tables

**Table 1:** Reported cases of synchronous malignant struma ovarii and thyroid carcinoma.

Reference	Patient Age (yrs)	Ovarian Mass Size (cm)	MSO Size (cm)	MSO Pathology	MSO Stage	Thyroid Carcinoma Size (cm), Type	ONCOGENIC EXPRESSION
Boyd et al [15]	30	8.5x5x4	-* *-* *-* *-	Papillary	No surface involvement, No LV invasion	1.0, Papillary, NO ETE	-* *-* *-* *-* *-* *-* *-

Brusca et al. [14]	30	7.3x3	0.9	Papillary	-* *-* *-* *-* *-* *-	0.2, Papillary, No ETE	-* *-* *-* *-* *-* *-* *-* *-

Janszen et al. [7]	52	10x8	-* *-* *-* *-	Follicular	-* *-* *-* *-* *-* *-* *-	0.3, Papillary, No ETE	-* *-* *-* *-* *-* *-

Catalina et al. [9]	33	9.5x7.5x3.5	2.5	Papillary	No surface involvement, No LV invasion	1.4, Papillary With ETE, Metastatic to lymph nodes	-* *-* *-* *-* *-

Leong et al. [8]	42	13.5	-* *-* *-* *-	Papillary	No surface involvement, No LV invasion	0.6-0.8, Papillary, bilateral multifocal, with ETE, mets to lymph nodes	BRAF – K-RAS – RET/PTC -

Marti et al. [2]	44	2.4	-* *-* *-* *-* *-	Papillary	-* *-* *-* *-* *-* *-* *-* *-	0.5, Papillary with ETE, mets to lymph nodes	BRAF -

Middelbeek et al [16]	55		1.5	Papillary	No surface involvement, No LV invasion	1.2, Papillary, No ETE	BRAF -

Capitato et al. [17]	35	8.2x7x6		Papillary	No surface involvement	1.7, Papillary, No ETE	BRAF -

Krishnamurthy et al. [18]	51	13x7.6	0.8	Papillary		0.4, papillary follicular variant	-* *-* *-* *-

Lim et al. [19]	63	3x2.5x1.5	-* *-* *-* *-* *-	Papillary	No surface involvment, No LV invasion	Papillary, mets to lymph nodes	-* *-* *-* *-

Present case	30	12.9	6.0	Papillary	No surface involvment, LV invasion	0.6, Papillary, with ETE, mets to lymph nodes	BRAF – RET/PTC – TERT -

Abbreviations: MSO, malignant struma ovarii; LV, lymphovascular; ETE, extrathyroid extension.

## Abbreviations

MSO: Malignant struma ovarii
RAI: Radioactive iodine
TSH: Thyroid stimulating hormone
FT4: Free thyroxine
FT3: Free triiodothyronine
WBS: Whole body scan
SPECT/CT: Single-photon emission computer tomography/computer tomography
LV: Lymphovascular
ETE: Extrathyroid extension.
